# The Variation and Influencing Factors of Volatile Organic Compounds on Branch and Leaf of *Phoebe hui* W.C. Cheng ex Yen C. Yang

**DOI:** 10.3390/life16071072

**Published:** 2026-06-26

**Authors:** Jiayi Wu, Jianghong Qian, Ruixian Zheng, Yunjie Gu, Jian Peng, Hongying Guo, Suyuan Zhang, Ruiqi Wang, Yulin Wei, Minhao Liu, Yi Wang, Jinwu Li, Lianghua Chen, Hanbo Yang

**Affiliations:** 1 Ecology and Conservation in the Upper Reaches of the Yangtze River Key Laboratory of Sichuan Province, Sichuan Mt. Emei Forest Ecosystem National Observation and Research Station, College of Forestry, Sichuan Agricultural University, Chengdu 611130, China; 13567615022@163.com (J.W.); 18183660294@163.com (J.Q.); zhengruixian_1106@163.com (R.Z.); z59951883@163.com (S.Z.); ruiqiwang004@gmail.com (R.W.); 13178392079@163.com (Y.W.); chenlh@sicau.edu.cn (L.C.); 2 Ecological Conservation, Restoration and Resource Utilization in Forest and Wetland Key Laboratory of Sichuan Province, Sichuan Academy of Forestry, Chengdu 610081, China; pjsclky432@163.com (J.P.); zhongli6@126.com (H.G.); sclkylmh@163.com (M.L.); x_wangyier@163.com (Y.W.); 13880690362@163.com (J.L.)

**Keywords:** *Phoebe hui*, branch, leaf, volatile organic compounds, environmental factors

## Abstract

Plant volatile organic compounds (VOCs) are crucial for communication, defense, pollination, and environmental adaptation, playing a key role in the survival and interaction of plants within ecosystems. However, as an important tree species rich in VOCs, the variation characteristics and influencing factors of VOCs on *Phoebe hui* W.C. Cheng ex Yen C. Yang remain unclear. In this study, we identified 106 VOCs across branches and leaves, of which 91 and 56 were detected, respectively, with 41 in both tissues. Sesquiterpenoids, olefins, fatty acids and conjugates dominated the VOC profiles of branches and leaves. Branches showed higher accumulation of sesquiterpenoids, fatty acids and conjugates, whereas leaves were enriched in olefins. The VOCs distribution and accumulation were strongly structured by environmental variation, with mean annual precipitation (MAP) and soil organic carbon (SOC) emerging as the primary drivers. Together, these findings elucidate the variation patterns and environmental determinants of VOCs in *P. hui*, providing a foundation for resource conservation and utilization while informing studies of VOC diversity across *Phoebe* species.

## 1. Introduction

Volatile organic compounds (VOCs) are key plant secondary metabolites involved in growth, development, stress responses, inter- and intraspecific communication, and attracting pollinators and herbivore predators [1,2]. Notably, their aromatic properties often link ecological functions with characteristic fragrances. In Lauraceae, VOCs underpin species-specific aromatic profiles, serving as ecological mediators and indicators of economic value, with application in spices production, cosmetics, and ecological conservation [3,4]. An investigation into VOCs of *Anthemis cotula* L. identified 167 VOCs, with terpenoids, particularly sesquiterpenes and monoterpenes, being dominant. Significant variations in VOC composition were observed across different seasons, plant organs, and altitudes [5]. Urban plants largely emit isoprene, monoterpenes, and sesquiterpenes, influenced by temperature, humidity, and light [6]. A total of 77 VOCs were identified in tomatoes, primarily consisting of monoterpenes and sesquiterpenes. Geographic barriers were found to drive ecological differences in VOC composition [7].

VOCs are affected by multiple factors, including genetic background, organ differentiation, developmental stage, and environmental conditions [8,9]. The major VOCs in *Teucrium polium* L. were α-Guaiene, trans-Caryophyllene, and γ-Elemene, with partial differences in VOCs possibly attributed to genetic variations among the plant materials or different geographical origins [10]. Functional differentiation among plant organs is a major intrinsic driver of VOC variation, as organ-specific physiological roles shape secondary metabolic regulation and give rise to distinct VOC profiles [5,11]. Environmental factors are key drivers that regulate the synthesis and accumulation of VOCs, often exerting compound-specific effects on both the presence/absence and relative content of individual VOCs. For instance, higher mean annual temperature (MAT) can promote monoterpenoid accumulation, such as limonene and α-pinene, while excessive heat may reduce the emission of thermally sensitive sesquiterpenes (e.g., β-caryophyllene) [12,13,14,15]. VOC profiles are also shaped by environmental conditions. Drought stress often induces the accumulation of long-chain alkanes, such as heptacosane and nonacosane, which serve as adaptive barriers against water loss, whereas high-precipitation regimes favor the production of leaf volatiles, such as (Z)-3-hexenyl acetate, by maintaining optimal substrate availability [16]. Soil organic carbon (SOC) can modulate plant carbon–nitrogen balance and shikimate pathway activity, thereby influencing the biosynthesis of volatile benzenoids and phenylpropanoids in forest ecosystems [17]. Soil pH could indirectly shape the roots and leaf of VOC profiles by regulating nutrient solubility and rhizosphere microbial communities [9]. Furthermore, geographical factors such as latitude and altitude shape regional microclimatic gradients, driving the floral volatile emissions pattern across plant families [18,19,20]. Within the *Phoebe* genus, tree age significantly affects the content of essential oil and fragrance components [21]. The environmental heterogeneity causes metabolic variation in *P. bournei* wood across regions [22].

*Phoebe hui* W.C. Cheng ex Yen C. Yang, an endangered, endemic, and precious tree species of Lauraceae, is mainly distributed in the subtropical evergreen broad-leaved forest areas of Sichuan Province, China [23]. Characterized by a straight trunk, fine-textured wood, and distinctive aromatic profile, this species is valued as both a precious timber tree and an aromatic plant, with a rich and diverse VOC profile [23]. Currently, research on *P. hui* primarily focuses on the VOC components in the fragrance of wood. However, the variation and influencing factors of VOCs remains limited. Therefore, future research directions can be developed in two aspects: on the one hand, a comparative analysis of VOC components in different parts of *P. hui* can be conducted to explore the relationship between composition and function, and on the other hand, the influence of environmental factors and intrinsic genetic regulatory mechanisms on VOC formation can be examined to advance efficient artificial breeding [24]. Here, we characterized VOC composition and relative abundance across natural populations of *P. hui* and identified the environmental drivers of VOC variation. The findings are expected to provide a scientific basis for *P. hui* germplasm conservation and to offer broader insights into VOC variation and ecological adaptation in *Phoebe* and other Lauraceae woody plants.

## 2. Materials and Methods

### 2.1. Test Materials

**Figure 1:**
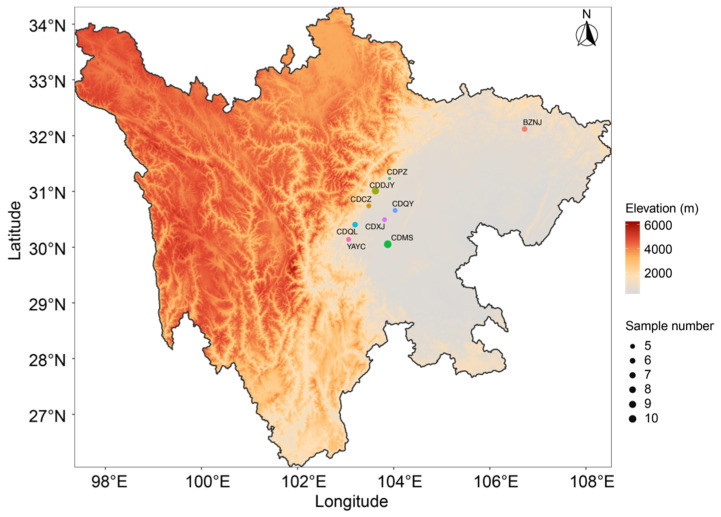
Information on sample collection of *P. hui* population. Note: The region is Sichuan, China. CDCZ: Chongzhou, Chengdu, Sichuan Province, China. CDQL: Qionglai, Chengdu, Sichuan Province, China. YAYC: Yucheng, Ya’an, Sichuan Province, China. BZNJ: Nanjiang, Bazhong, Sichuan Province, China. CDMS: Meishan, Chengdu, Sichuan Province, China. CDDJY: Dujiangyan, Chengdu, Sichuan Province, China. CDPZ: Pengzhou, Chengdu, Sichuan Province, China. CDQY: Qingyang, Chengdu, Sichuan Province, China. CDXJ: Xinjin, Chengdu, Sichuan Province, China.

A total of 55 individuals from 9 natural populations of *Phoebe hui* W.C. Cheng ex Yen C. Yang were sampled on 25 October 2024 (Figure 1, Table S1). Given the restricted distribution of *P. hui* and the small population sizes observed in the field, no population with more than 30 individuals was identified. Accordingly, all available individuals within each population were sampled, yielding 5–10 individuals per population. Healthy and mature branches and leaves were collected from each individual. Samples were transported to the laboratory, air-dried at room temperature, ground into powder, and sequentially passed through 100-mesh sieves prior to VOC extraction.

### 2.2. Volatile Organic Compounds (VOCs) Extraction and Gc-Ms Analysis

A total of 5 g of sample powder was transferred to an extraction vial preconditioned in the gas chromatograph injection port at 250 °C for 120 min and preheated at 30 °C for 10 min. Headspace adsorption was performed using a 100 µm polydimethylsiloxane (PDMS) fiber positioned 0.5 cm above the powder at 30 °C for 30 min. The extraction fibers were subjected to GC-MS desorption for 3 min (at 250 °C) to perform GC-MS analysis [21].

The VOCs were analyzed using a SHIMADZU GCMS-QP2010 Ultra instrument (SHIMADZU, Kyoto, Japan). The chromatographic column was an RXI-5SILMS (30 m × 0.25 mm, Restek Corporation, Bellefonte, PA, USA). The temperature program employed for VOCs detection had the following settings: an initial temperature of 60 °C, an increase to 180 °C at 8 °C/min, and then an increase to 220 °C at 10 °C/min. The ion source was 230 °C.

### 2.3. Relative Composition Analysis of Metabolites

The VOCs were qualitatively analyzed using tandem searches with the NIST library [25] and retention index matching [26,27] in the METLIN [28], MassBank [29], LIPIDMAPS [30], and Golm Metabolome Database [31]. For quantitative analysis, a normalization method was used based on the chromatographic peak area. The relative content of each VOC was represented by the percentage of its peak area relative to the total peak area [32].

### 2.4. Environmental Data Acquisition

The annual mean temperature (MAT), mean diurnal range (MDR), isothermality (ISO), temperature seasonality (TS), maximum temperature of the warmest month (MTWM), minimum temperature of the coldest month (MTCM), temperature annual range (TAR), mean temperature of the wettest quarter (MTWQ), mean temperature of the driest quarter (MTDQ), mean temperature of the warmest quarter (MTWAQ), mean temperature of the coldest quarter (MTCQ), annual precipitation (MAP), precipitation of the wettest month (PWM), precipitation of the driest month (PDM), precipitation seasonality (coefficient of variation, PS), precipitation of the wettest quarter (PWQ), precipitation of the driest quarter (PDQ), precipitation of the warmest quarter (PWAQ), and precipitation of the coldest quarter (PCQ) were obtained from the WorldClim database (http://www.worldclim.org/) [33]. These data included 19 climate variables with a spatial resolution of 2.5 arc-minutes. Additionally, soil organic carbon (SOC) content and soil pH data were obtained from the Harmonized World Soil Database (HWSD) [34] with a spatial resolution of 1.0 km. Environmental factors of latitude, longitude, altitude, climate, and soil variables, were analyzed using ‘readxl’, ‘psych’, ‘caret’, and ‘usdm’ packages in R. These factors were sequentially screened based on their coefficient of variation (CV > 5%). Correlation analysis based on an *r* = 0.7 threshold and multicollinearity verification using the variance inflation factor (VIF < 10) were conducted. After performing collinearity tests and correlation analyses, two climate variables, annual mean temperature (MAT) and annual precipitation (MAP), were selected for further analysis (Table S2).

### 2.5. Data Analysis

The VOCs identified in branch and leaf samples were classified and annotated against the HMDB database. Data visualization, missing value analysis, and format standardization were performed using the ‘corrplot’, ‘naniar’, and ‘dplyr’ packages in R. The standardized environmental factor dataset was then exported after screening. Principal component analysis (PCA) of VOCs in branches, leaves, and branches + leaves was performed and visualized using the ‘stats’ and ‘factoextra’ package in R. MetaboAnalyst was used for hierarchical clustering analysis (HCA) and K-means clustering analysis to examine the accumulation patterns of VOCs, as well as for VOC enrichment analysis [35]. Redundancy analysis (RDA) was performed using the ‘vegan’ package in R to examine the impact of environmental factors on VOCs, and visualizations were created using ‘ggplot2’in R [36].

## 3. Results

### 3.1. Volatile Organic Compounds (VOCs) Components in P. hui

**Figure 2:**
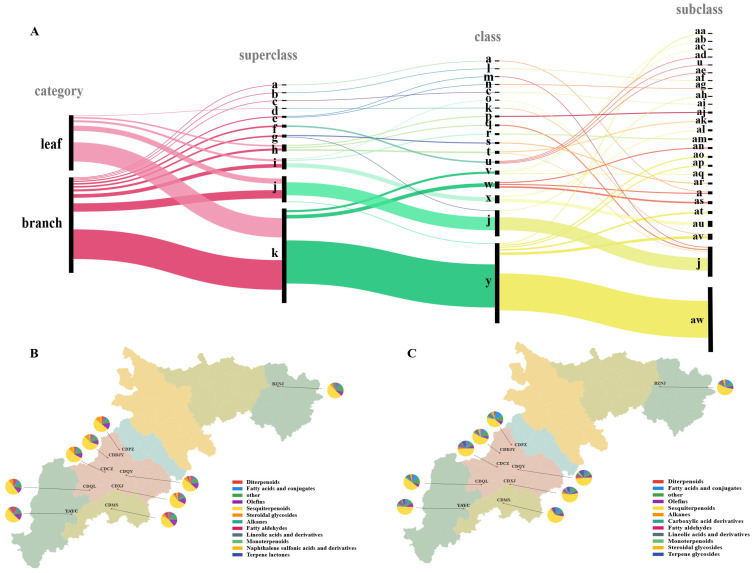
VOC components in the branches and leaves of *P. hui* and their geographic distribution. (**A**) The VOC components of branches and leaves in *P. hui* natural populations. (**B**) The VOC components in leaves at the nine populations. (**C**) The VOC components in branches at the nine populations. Note: In (**A**), label ‘a’ refers to linoleic acids and derivatives, ‘b’ to organic nitrogen compounds, ‘c’ to tetrapyrroles and derivatives, ‘d’ to organic compounds, ‘e’ to organo-heterocyclic compounds, ‘f’ to organic acids and derivatives, ‘g’ to phenylpropanoids and polyketides, ‘h’ to benzenoids, ‘i’ to hydrocarbons, ‘j’ to other compounds, ‘k’ to lipids and lipid-like molecules, ‘l’ to organonitrogen compounds, ‘m’ to benzisoxazoles, ‘n’ to furans, ‘o’ to polycyclic hydrocarbons, ‘p’ to benzene and substituted derivatives, ‘q’ to fluorenes, ‘r’ to unsaturated hydrocarbons, ‘s’ to macrolides and analogues, ‘t’ to naphthalenes, ‘u’ to carboxylic acids and derivatives, ‘v’ to steroids and steroid derivatives, ‘w’ to fatty acyls, ‘x’ to saturated hydrocarbons, ‘y’ for prenol lipids, ‘aa’ for triterpenoids, ‘ab’ for humulenol-II, ‘ac’ for vitamin D and derivatives, ‘ad’ for amino acids, peptides, and analogues, ‘ae’ for dicarboxylic acids and derivatives, ‘af’ for quaternary ammonium salts, ‘ag’ for furoic acid and derivatives, ‘ah’ for flavonoids, ‘ai’ for corrinoids, ‘aj’ for benzoic acids and derivatives, ‘ak’ for naphthalenesulfonic acids and derivatives, ‘al’ for cycloalkanes, ‘am’ for olefins, ‘an’ for fatty aldehydes, ‘ao’ for terpene glycosides, ‘ap’ for terpene lactones, ‘aq’ for steroidal glycosides, ‘ar’ for zearalenones, ‘as’ for fatty acids and conjugates, ‘at’ for diterpenoids, ‘au’ for alkanes, ‘av’ for monoterpenoids, and ‘aw’ for sesquiterpenoids. In (**B**,**C**), CDCZ: Chongzhou, Chengdu, Sichuan Province, China. CDQL: Qionglai, Chengdu, Sichuan Province, China. YAYC: Yucheng, Ya’an, Sichuan Province, China. BZNJ: Nanjiang, Bazhong, Sichuan Province, China. CDMS: Meishan, Chengdu, Sichuan Province, China. CDDJY: Dujiangyan, Chengdu, Sichuan Province, China. CDPZ: Pengzhou, Chengdu, Sichuan Province, China. CDQY: Qingyang, Chengdu, Sichuan Province, China. CDXJ: Xinjin, Chengdu, Sichuan Province, China.

A total of 106 VOCs were identified in both the branch and leaf of *P. hui* (Figure 2A, Table S3). Among these, 91 and 56 VOCs were detected in the branch and leaf samples, respectively, with 41 VOCs shared between both branches and leaves (Figure S1, Table S4). The 106 VOCs were annotated to 11 superclasses, 16 classes, and 18 subclasses. At the superclass level, lipids and lipid-like molecules (71), hydrocarbons (9), and benzenoids (9) were the dominant groups. Branches contained nine superclasses, and leaves contained five, with three shared between tissues. At the class level, the VOCs were mainly represented by prenol lipids (34). Branches and leaves contained 14 and 10 classes, respectively, including eight shared classes, such as prenol lipids, unsaturated hydrocarbons, and fatty acyls. At the subclass level, sesquiterpenoids (46), olefin (1), fatty acids and conjugates (3) were the main groups. Branches contained all 18 subclasses, whereas leaves contained 11, all of which were shared with branches. Seven subclasses, including terpene glycosides, carboxylic acid derivatives, and cycloalkanes, were branch-specific. Overall, branches exhibited greater VOC diversity than leaves across all classification levels, indicating a more complex VOC profile in *P. hui* branches. Analysis of VOCs in branches and leaves from nine sampling sites (Figure 2B,C) reveals that diterpenoids, olefins, fatty acids and conjugates have relatively low proportions with minimal regional differences. Diterpenoids and olefins are generally higher in leaves than in branches, while fatty acids and conjugates are more abundant in branches. Sesquiterpenoids show high overall proportions and exhibit distinct regional variations: in branches, Meishan (CDMS) has the highest proportion (60.94%) and Pengzhou (CDPZ) the lowest (40.75%); in leaves, Dujiangyan (CDDJY) has the highest proportion (57.09%) and Meishan (CDMS) the lowest (43.64%).

### 3.2. Differential Accumulation of VOCs

**Figure 3:**
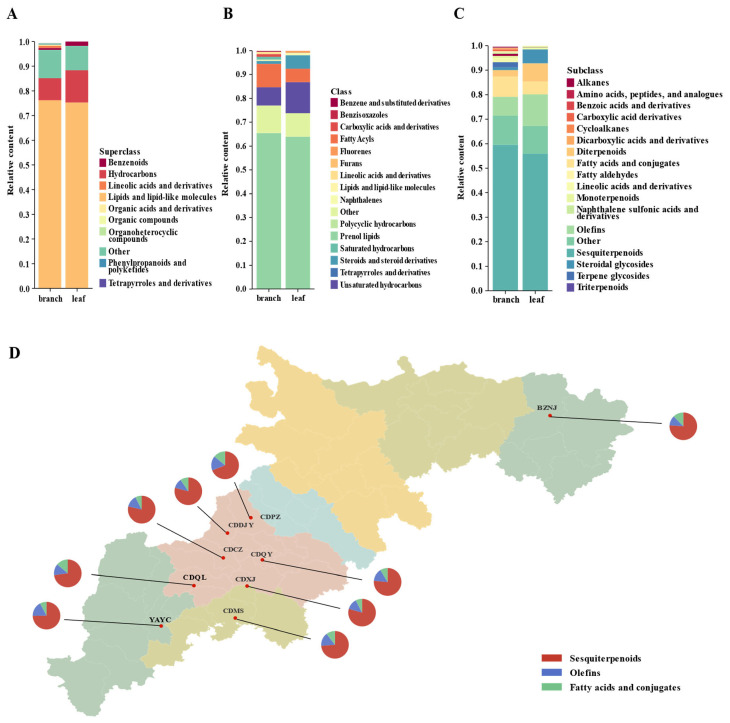
Comparison of VOC components in the branches and leaves of *P. hui* and their geographic distribution at the subclass level. (**A**–**C**) Comparative analysis of the relative content of VOCs between the branches and leaves. (**D**) The proportions of the top three VOCs at the subclass level across different regions. Note: In (**D**), CDCZ: Chongzhou, Chengdu, Sichuan Province, China. CDQL: Qionglai, Chengdu, Sichuan Province, China. YAYC: Yucheng, Ya’an, Sichuan Province, China. BZNJ: Nanjiang, Bazhong, Sichuan Province, China. CDMS: Meishan, Chengdu, Sichuan Province, China. CDDJY: Dujiangyan, Chengdu, Sichuan Province, China. CDPZ: Pengzhou, Chengdu, Sichuan Province, China. CDQY: Qingyang, Chengdu, Sichuan Province, China. CDXJ: Xinjin, Chengdu, Sichuan Province, China.

At the superclass level, VOC profiles in both branches and leaves were dominated by lipids and lipid-like molecules, accounting for 76.19% and 75.23% of the total, respectively (Figure 3A). At the class level, prenol lipids (64.64%), unsaturated hydrocarbons (10.32%), and fatty acyls (7.71%) represent the predominant VOC classes in both branches and leaves (Figure 3B). Branches showed higher relative abundances of prenol lipids and fatty acyls but markedly lower levels of unsaturated hydrocarbons than leaves. At the subclass level, sesquiterpenoids (57.76%), olefins (10.31%), fatty acids and conjugates (6.69%) represented the predominant VOC subclasses in both branches and leaves (Figure 3C). The relative contents of sesquiterpenoids, fatty acids and their conjugates in branches were higher than those in leaves; however, the relative content of olefins in branches was significantly lower than that in leaves. VOCs differed markedly between branches and leaves, with leaves enriched in volatile terpenes and branches characterized by structural protective metabolites such as alkanes, reflecting organ functional specialization and differential environmental adaptation. Analysis of VOCs across regions (Figure 3D) shows that sesquiterpenoids are the dominant components, with proportions significantly higher than other VOCs. The highest proportion is observed in Xinjin (CDXJ) and the lowest in Pengzhou (CDPZ). Fatty acids and conjugates, as well as olefins, show minimal variations in most regions, but significant differences are seen in Ya’an (YAYC) and Chongzhou (CDCZ).

### 3.3. Principal Component and Clustering Analysis

**Figure 4:**
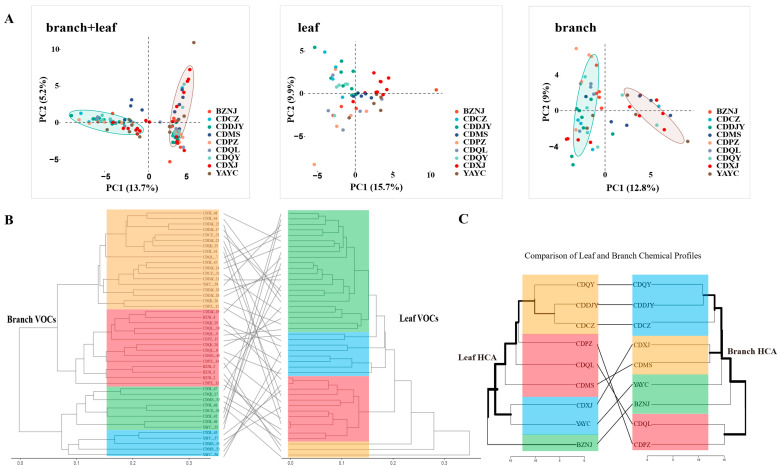
Principal component analysis and cluster analysis of VOCs in the branches and leaves of *P. hui.* (**A**) Principal component analysis based on VOCs database. (**B**,**C**) Cluster analysis of metabolites in the branches and leaves of *P. hui*. Note: The same color represents the same group. CDCZ: Chongzhou, Chengdu, Sichuan Province, China. CDQL: Qionglai, Chengdu, Sichuan Province, China. YAYC: Yucheng, Ya’an, Sichuan Province, China. BZNJ: Nanjiang, Bazhong, Sichuan Province, China. CDMS: Meishan, Chengdu, Sichuan Province, China. CDDJY: Dujiangyan, Chengdu, Sichuan Province, China. CDPZ: Pengzhou, Chengdu, Sichuan Province, China. CDQY: Qingyang, Chengdu, Sichuan Province, China. CDXJ: Xinjin, Chengdu, Sichuan Province, China.

To further investigate interpopulation variation in VOC profiles, principal component analysis (PCA) revealed δ-cadinene, neophytadiene, and phytol as the major contributors to PC1, reflecting variation in plant-derived terpenes components. In contrast, PC2 was mainly driven by caryophyllene, humulene, and α-eudesmol terpenes associated with plant defense and aroma (Figure 4A). Samples were distinctly separated into branch and leaf groups. The PCA based on leaf VOCs showed contribution rates of 15.69% and 9.90% to PC1 and PC2, with a cumulative variance of 25.60%, but no obvious differentiation among populations. The PCA-based branch VOCs showed contribution rates of 12.78% and 8.99% to PC1 and PC2, with a cumulative variance of 21.77%, of which, PC1 separated individuals by geographic origin, with Qionglai (CDQL) and Dujiangyan (CDDJY) samples clustering on the side of PC1 < 0 and Meishan (CDMS) and Xinjin (CDXJ), Chengdu samples on the PC1 > 0 side. The cumulative variance in the principal component analysis was relatively low, mainly due to the large number of analytical indicators and the substantial variation of each metabolite across different samples, which diluted the cumulative variance. However, the aim of our study is to compare the differences between samples based on all metabolites, so such a cumulative variance is acceptable.

The sesquiterpenes, hydrocarbons, and terpene alcohols were the main components of VOCs in *P. hui* branches and leaves. The key compounds included neophytadiene, phytol, copaene, germacrene D, γ-muurolene, cis-calamenene, cubenol, τ-cadinol, eudesmol, and n-hexadecanoic acid, driving clustering differentiation. Hierarchical clustering analysis (HCA) of the key VOCs showed hierarchical differentiation but no stable clustering by geographic population (Figure 4B,C). Branch samples were intermixed across clusters, suggesting weak correspondence between VOC profiles and population origin. Similarly, leaf samples formed small subgroups at high similarity levels but remained distributed across clusters, suggesting strong individual variation and environmental modulation of VOCs accumulation. K-means cluster analysis grouped branch individuals into four clusters, with populations dispersed across clusters, of which, cluster 1 included Qingyang (CDQY), Chongzhou (CDCZ) and Dujiangyan (CDDJY) individuals, cluster 2 included Xinjin (CDXJ) and Meishan (CDMS) individuals, cluster 3 included Ya’an (YAYC) and Bazhong (BZNJ) individuals, and cluster 4 included Qionglai (CDQL) and Pengzhou (CDPZ) individuals. Leaf VOCs also formed four clusters with different compositions; cluster 1 included Qingyang (CDQY), Dujiangyan (CDDJY) and Chongzhou (CDCZ) individuals, cluster 2 included Pengzhou (CDPZ), Qionglai (CDQL) and Meishan (CDMS) individuals, cluster 3 included Xinjin (CDXJ) and Ya’an (YAYC) individuals, and cluster 4 included Bazhong (BZNJ) individuals. The results of variance analysis for VOCs in leaves and branches across different regions are listed in Tables S5 and S6. Branch and leaf samples were highly intermixed across clusters, suggesting that the VOC profiles of *P. hui* were only weakly associated with population origin (Tables S5 and S6). The concordant results of PCA, HCA, and K-means clustering indicate that VOC accumulation in *P. hui* is more strongly associated with environmental factors and organs than with population origin or phylogenetic relationships.

### 3.4. Enrichment Analysis of VOCs

**Figure 5:**
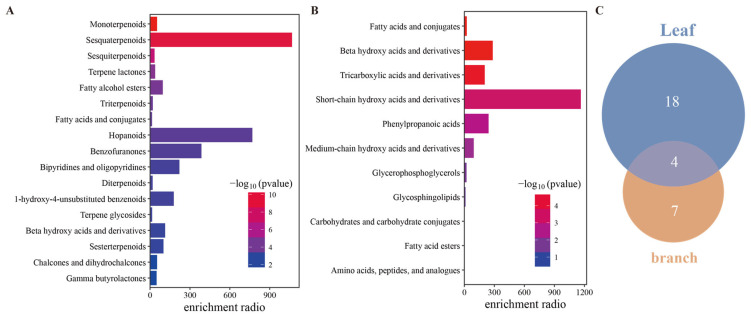
Enrichment pathway analysis of VOCs of *P. hui*. (**A**,**B**) Enrichment analysis of secondary metabolites in the leaves (**A**) and branches (**B**) of *P. hui*. (**C**) Venn diagram of metabolic pathways of branches and leaves.

KEGG enrichment analysis revealed a total of 29 pathways, among which four enriched pathways were shared between branches and leaves (Figure 5). In the branches, seven pathways were uniquely enriched. Among these, tricarboxylic acids and their derivatives demonstrated strong enrichment, involving 57 VOCs and reaching an extremely high level of significance (raw *p* = 4.55 × 10^−5^, FDR = 0.00809). Short-chain hydroxy acids and their derivatives included five VOCs, with one VOC detected. Additionally, fatty acids and their conjugates were significantly enriched in branches, showing higher significance compared to leaves. A total of 18 uniquely enriched pathways were identified in the leaves. Among these, monoterpenoids displayed the most significant enrichment, involving 61 VOCs with extremely high significance (raw *p* = 7.76 × 10^−11^, FDR = 6.91 × 10^−8^). Sesquiterpenoids included 68 VOCs and also showed highly significant enrichment. Furthermore, terpene lactones involved thirty-six VOCs, with three VOCs detected. In summary, leaves were uniquely enriched with monoterpenoids, sesquiterpenoids, and terpene lactones, which are closely related to their roles in photosynthesis and stress defense. In contrast, branches were specifically enriched for tricarboxylic acid (TCA) cycle-related substances, phenylpropanoic acids, and short-chain hydroxy acids, highlighting their involvement in steroid metabolism and energy cycling. This suggests that branches are functionally important for structural support and internal signal regulation.

### 3.5. The Main Environmental Factors’ Influence on VOC Accumulation

**Figure 6:**
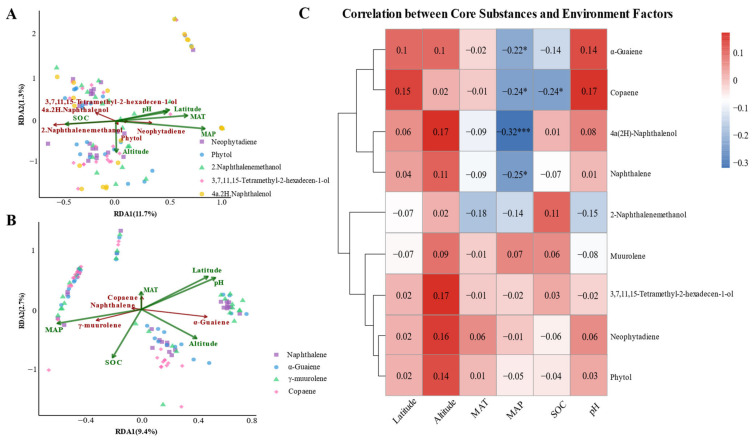
Relationship between VOCs in the branches and leaves of *P. hui* and environmental factors. (**A**,**B**) Analysis of the main environmental factors affecting the metabolism of branches (**A**) and leaves (**B**) of *P. hui*. (**C**) Correlation and significance analysis between the content of main internal compounds in leaves and environmental factors. Note: ‘*’ indicates significance, ‘*’ for *p* ≤ 0.05, and ‘***’ for *p* ≤ 0.001. The environment factors shown in (**C**) include latitude, altitude, MAT, MAP, SOC, and pH. MAT: mean annual temperature. MAP: mean annual precipitation. SOC: soil organic carbon.

Redundancy analysis (RDA) was performed to clarify the environmental drivers of the key VOCs in branches and leaves (Figure 6A,B). The results showed that neophytadiene and 3,7,11,15-tetramethyl-2-hexadecen-1-ol correlated strongly with pH, latitude, mean annual temperature (MAT), and precipitation (MAP); naphthalene derivatives aligned with soil organic carbon (SOC); and phytol with altitude. MAT, latitude, and pH primarily influenced VOC variation along one axis, while altitude and SOC affected another, indicating multidimensional environmental impacts. Although RDA explained a relatively small proportion of sesquiterpene variation, naphthalene, α-guaiene, and γ-muurolene were associated with MAP, SOC, and altitude, suggesting joint effects of these factors on their synthesis and accumulation. The accumulation of copaene related to latitude and MAT, implying climatic and photoperiod influences. Although RDA suggested that latitude and MAT may structure VOC variation along one ordination axis, formal correlation analysis identified MAP and SOC as statistically significant environmental predictors of key VOC accumulation in *P. hui*, while geographic location and MAT showed no significant individual effects. These results are consistent with the primary drivers reported in the Abstract.

The results of correlation analysis showed that latitude, altitude, and MAT had no significant effects on neophytadiene, naphthalene derivatives, phytol, and copaene (Figure 6C). In contrast, MAP significantly inhibited copaene (*r* = −0.239, *p* = 0.014) and naphthalene derivatives (*r* = −0.249, *p* = 0.010). SOC also significantly suppressed copaene (*r* = −0.238, *p* = 0.014). Overall, MAP and SOC are the key factors negatively impacting the accumulation of the key VOCs in *P. hui*, while geographic location and MAT showed no significant impact.

## 4. Discussion

Studying plant volatile organic compounds (VOCs) is essential for understanding how plants interact with their environment, defend against threats, attract pollinators, and adapt to changing conditions [37,38]. This knowledge has significant implications for agriculture, ecosystem health, and the development of sustainable pest management strategies [39,40]. Due to the limitations of experimental techniques, we are currently unable to directly collect VOCs from live *P. hui* specimens for analysis. Therefore, we dried and ground the plant samples, using the powder for detection in order to identify the VOCs as comprehensively as possible. A total of 106 VOCs were identified in the branches and leaves of *P. hui*, including 91 VOCs in the branches and 56 VOCs in the leaves, with 41 VOCs shared between them. Branches exhibited greater VOC diversity than leaves, supporting functional specialization. Leaves were enriched in volatile terpenoids associated with photosynthesis and defense, whereas branches accumulated tricarboxylic acid cycle–related substances, phenylpropanoic acids, and short-chain hydroxy acid derivatives linked to energy cycling and structural support [41]. Not only are there differences in VOC composition between the branches and leaves of plants, but other parts also exhibit differences in VOC composition. After analyzing the VOCs of seeds and leaves in plants of the same genus as *Phoebe zhennan* and *Phoebe chekiangensis*, it was found that leaves are typically rich in terpenoids and GLV-related compounds. These compounds are often associated with anti-herbivory, allelopathy, or stress responses [42,43]. In contrast, seeds are rich in volatile substances characterized by fruity, sweet, and floral aromas, which may be related to seed protection and attracting dispersal agents [42,43]. The experiment showed that branches and leaves of *P. hui* from different natural distribution regions both contained sesquiterpenoids, olefins, fatty acids and conjugates. However, the VOC composition and content varied among regions, indicating that these differences can be effectively used for species classification. After analyzing the VOCs of different *Prunus salicina*, they can be classified into distinct groups, with significant differences in fruit flavor observed between the groups [44]. Similarly, the analysis of VOCs from different tea varieties has identified key VOCs influencing the classification of tea cultivars [45,46]. GC-MS and PCA identified nine core VOCs: neophytadiene, 3,7,11,15-tetramethyl-2-hexadecen-1-ol, phytol, 2-naphthalenemethanol, 4a,2H-naphthalenol, naphthalene, α-guaiene, γ-muurolene, and copaene. Based on these nine core VOCs, cluster analysis grouped the samples from nine natural locations into four distinct clusters. Samples from the same geographic location did not cluster together but were dispersed across multiple groups. This suggests that the metabolic accumulation patterns of *P. hui* might be significantly influenced by non-genetic factors such as environmental conditions and developmental stages.

Investigating the effects of environmental factors on VOC accumulation helps reveal how plants adapt to different habitats and explains geographic variation in chemical profiles [47]. It also provides insights into the ecological functions and biosynthetic regulation of volatile compounds [48]. Temperature and precipitation are major environmental factors influencing plant VOC biosynthesis and emission [49,50]. Temperature can regulate stomatal opening and thereby control VOC emissions [49]. It also affects the activity of many enzymes involved in VOC biosynthesis [49]. Under high-temperature conditions, plants may release VOCs to improve their photosynthetic efficiency [50]. Water availability can likewise influence VOC emissions by affecting stomatal opening; however, its inhibitory effect on terpene emission is usually only pronounced under severe drought conditions [50]. These observations suggest that temperature has a greater influence on VOCs than water availability [51]. Unlike previous studies emphasizing temperature, mean annual precipitation (MAP), rather than mean annual temperature (MAT), proved to be the dominant climatic driver, likely due to *P. hui*’s distribution in humid subtropical regions with stable hydrothermal conditions. Soil pH and soil organic carbon (SOC) can indirectly influence plant VOC emissions by altering nutrient availability, microbial communities, water retention, and plant physiological status [10,47,52]. These soil properties may therefore affect both the biosynthesis and accumulation patterns of VOCs [10,47,52]. This view is consistent with the results of our study: the accumulation of naphthalene derivatives and phytol are influenced by SOC, while neophytadiene is affected by soil pH. Future research integrating multi-omics and seasonal sampling is proposed to elucidate molecular mechanisms underpinning environment-driven metabolic variation and to aid in the targeted cultivation of aromatic germplasm with ecological and bioactive value.

## 5. Conclusions

This study systematically characterized VOCs in the branches and leaves of *P. hui* across nine natural distribution areas, revealing a total of 106 VOCs. PCA analysis identified nine core VOCs, including neophytadiene, 3,7,11,15-tetramethyl-2-hexadecen-1-ol, phytol, 2-naphthalenemethanol, 4a,2H-naphthalenol, naphthalene, α-guaiene, γ-muurolene, and copaene. KEGG analysis revealed twenty-nine metabolic pathways, seven and eighteen were found in branches and leaves, respectively, with four shared by both. The distribution and accumulation of VOCs were strongly influenced by environmental factors, particularly mean annual precipitation (MAP) and soil organic carbon (SOC). These findings enrich our understanding of VOC variation and its influencing factors in *Phoebe hui* and provide theoretical support for future studies on VOCs in related species within the same genus.

## Data Availability

Data will be made available on request.

## Supplementary Materials

The following supporting information can be downloaded at: https://www.mdpi.com/article/10.3390/life16071072/s1, Table S1: Information on sample collection from natural populations of *P. hui*; Table S2: Selection results of six environmental factors; Table S3: Complete information table of 106 VOCs; Table S4: Summary table of key VOCs in *P. hui*; Table S5: ANOVA and multiple comparisons (leaf); Table S6: ANOVA and multiple comparisons (branch); Figure S1: Key VOCs in branch and leaf of *P. hui*.
