# Supplementary figures and images for: The Variation and Influencing Factors of Volatile Organic Compounds on Branch and Leaf of Phoebe hui W.C. Cheng ex Yen C. Yang
Source: Life (Basel). 2026 Jun 26;16(7):1072. doi: 10.3390/life16071072 (PMC13412579; doi:10.3390/life16071072)

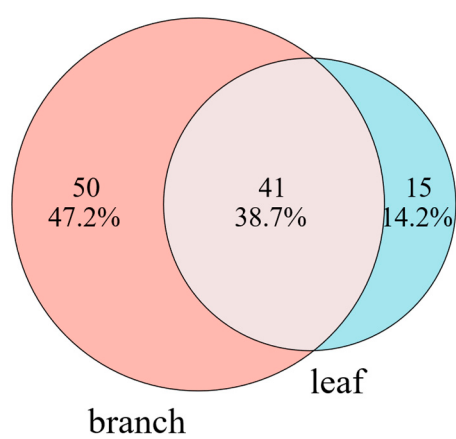

Figure S1 Key VOCs in branch and leaf of *Phoebe hui*

Supplement: Supplementary file 1 [file life-16-01072-s001.zip › Figure.pdf]
